# Coral-Associated Bacteria Provide Alternative Nitrogen Source for Symbiodiniaceae Growth in Oligotrophic Environment

**DOI:** 10.3390/microorganisms13040748

**Published:** 2025-03-26

**Authors:** Yawen Liu, Yanying Hua, Yan Yi, Jicai Liu, Pengcheng Fu

**Affiliations:** 1 School of Life and Pharmaceutical Sciences, Hainan University, 58 Renmin Avenue, Haikou 570228, China; 21110832000005@hainanu.edu.cn (Y.L.); 22220951350025@hainanu.edu.cn (Y.H.); 23210707030007@hainanu.edu.cn (Y.Y.); 23220951340128@hainanu.edu.cn (J.L.); 2 State Key Laboratory of Marine Resource Utilization in South China Sea, Hainan University, 58 Renmin Avenue, Haikou 570228, China

**Keywords:** coral-associated bacteria, nitrogen source, organic compounds, Symbiodiniaceae, trophallaxis

## Abstract

Coral reefs thrive in nutrients-poor waters, and their survival strategy in such oligotrophic marine environments remains largely unexplored. Current coral research has focused on the interplay between the animal hosts, symbiotic Symbiodiniaceae, and associated bacteria, with little attention given to their individual interactions. Here, we integrated biochemical, transcriptomic, and metabonomic analyses of the clade D Symbiodiniaceae strain AG11 to investigate the growth-assisting mechanisms of symbiotic bacteria. Our findings indicate that metabolic trophallaxis between Symbiodiniaceae and symbiotic bacteria plays a crucial role in enhancing survival and population growth under nitrogen-depleted conditions, commonly found in typical coral habitats. Notably, the exchange of organic compounds between Symbiodiniaceae and bacteria significantly boosts nitrogen uptake in their free-living state. Furthermore, we demonstrated how beneficial bacteria influence the survival of Symbiodiniaceae in response to environmental changes, which are vital for coping with nitrogen-depleted conditions where coral reefs are particularly vulnerable.

## 1. Introduction

Reef-building corals embody a complex symbiosis, involving intricate interactions between cnidarians and diverse microbial partners. The scarcity of inorganic nutrients in coral reef ecosystems highlights the critical role of nutrient exchange among coral polyps, Symbiodiniaceae, and bacterial communities. This intricate exchange supports coral growth and development, ultimately determining the ecological success and resilience of the entire coral symbiosis. Symbiodiniaceae (commonly known as zooxanthellae) are the photosynthetic symbionts living within coral tissues, relying on external sources of nitrogen to support their cell growth and metabolism. For coral reproduction, adult or larvals must acquire Symbiodiniaceae cells from the environment or from their parents [1]. Additional, coral-derived viable Symbiodiniaceae possess the capability to horizontally infect other coral individuals or larval, thereby fostering the formation of novel symbiotic associations [2]. Consequently, the survival strategy of both free-living and symbiotic Symbiodiniaceae cells is crucial for maintaining healthy coral communities.

The symbiotic association of Symbiodiniaceae and bacteria in the coral holobiont was explored by earlier research in the literature, which elucidated that algal photosynthesis provides oxygen and organic matters for coral and associated prokaryotic microorganisms to metabolize in both respiration and biosynthesis [3,4]. On the other hand, bacteria are capable of provisioning vitamin B_12_ [5], carotenoids [6], and auxins to Symbiodiniaceae. This is exemplified in the sulfur cycling [7], carbon cycling [8], and nitrogen cycling [9] processes that involve interactions between the symbiotic organisms. Nitrogen-fixing bacteria are capable of converting atmospheric nitrogen into biologically available ammonia, and they can enhance nitrogen fixation rates to sustain the productivity of coral holobionts [10]. Therefore, the study on the Symbiodiniaceae-bacteria interactions is a logical step toward understanding the mechanism underlying the intricate multi-partner interactions within the coral holobiont.

Nitrate is one of the key nitrogen sources in the marine environment. The density of Symbiodiniaceae has been observed to positively correlate with seawater nitrate concentrations [11,12]. The presence of diazotrophs enhances the adaptability of coral holobionts to nitrogen-limited environments, thereby contributing to the maintenance of productivity and stability in coral reef ecosystems. The metabolism of nitrogen and carbon is intimately related, with reduced nitrogen supply often leading to a decrease in photosynthetic proteins, which, in turn, diminishes carbon assimilation. BMC for corals play a crucial role in promoting the growth of Symbiodiniaceae under nitrogen stress conditions, accomplishing this through a diverse array of compensatory mechanisms [13]. Some critical questions arise: Do free-living or symbiotic Symbiodiniaceae survive exclusively through photosynthesis in oligotrophic environments? If not, does the symbiotic bacteria union enable Symbiodiniaceae to survive in nitrogen-depleted conditions? In addition, similar to acquiring nitrogen from inorganic nitrogen or dissolved organic nitrogen, do Symbiodiniaceae also derive nitrogen from bacterial partners?

In this study, we integrate biochemical, transcriptomic, and metabonomic analyses of Symbiodiniaceae of the clade D strain AG11 (designated AG11 throughout) to investigate the growth-assisting mechanisms of symbiotic bacteria on Symbiodiniaceae. Our objective was to demonstrate that beneficial microorganisms for corals (BMC) can significantly enhance the growth and survival of Symbiodiniaceae by providing alternative nitrogen source through various compensatory mechanisms even under nitrogen-limited environmental conditions. These mechanisms include facilitating nitrogen acquisition, providing essential metabolites, and reducing oxidative stress, thereby maintaining the health and metabolic efficiency of Symbiodiniaceae. However, it is important to note that not all potential symbiotic bacteria possess the ability to promote the growth of Symbiodiniaceae, indicating a specificity in their interactions. Our findings revealed that Symbiodiniaceae can sustain healthy growth under nitrogen-limited conditions by obtaining alternative nitrogen sources and nutrients provided by beneficial bacteria. This provides meaningful insights into microbial contributions to Symbiodiniaceae success and coral resilience in oligotrophic marine environments.

## 2. Methods

### 2.1. Isolation of Bacterial Strains and Symbiodiniaceae from Corals

Two coral samples, *Acropora hyacinthus* and *Galaxea fascicularis*, were collected from Wuzhizhou, Hainan island, China (18°18′52.8″ N 109°46′07.9″ E). The samples were put in sterile plastic bags inside Styrofoam boxes containing 1 L of seawater and transported to the laboratory. Upon arrival, the coral tissues were cut into 5 cm pieces using pliers (Maxspect, Ltd., Hongkong, China).

The coral surface was rinsed three times with sterile seawater. The cleaned coral fragments were put in a mortar and ground with sterile seawater until completely homogenized, then transferred to a 50 mL tube and centrifuged at 5000× *g* for 5 min. Triplicate samples (50–100 μL) were seeded onto petri dishes containing 20 mL of a Marine Agar 2216E medium and an L1 medium (see Tables S1 and S2) with a group of antibiotics (final concentration of 50 mg L^−1^ ampicillin, kanamycin, chloramphenicol, chlortetracycline, streptomycin, and penicillin) [14]. The centrifuged pellet was re-suspended in sterile seawater, and serial dilutions (10^−1^, 10^−2^, 10^−3^, 10^−4^, and 10^−5^) were seeded onto the above medium.

All plates were incubated at 26 °C for 72 h, with a 12 h day/12 h night cycles (lighting intensity: 60 μmol of photons m^−2^ s^−1^ from 07:00 to 19:00). Morphologically different single colonies were isolated and stored in 15% glycerol stocks at −80 °C for use in the future.

### 2.2. Identification of Bacteria with 16S rDNA and Symbiodiniaceae with ITS2 Sequence

For the identification of isolated bacteria, isolates were cultured with single colonies in an MA2216E medium overnight, and cells were centrifuged at 10,000× *g* for 5 min. Bacterial genomes were isolated using a TIANamp Bacteria DNA Kit (TianGen Biotech Co., Beijing, China) following the manufacturer’s user manual. The DNA concentration was measured using a micro ultraviolet spectrophotometer (Nanodrop-2000, Thermo Fisher, Waltham, MA, USA). See Table S3 for target gene primers and PCR cycle information.

Based on the required biomass, Symbiodiniaceae cultures were cultured in 6-well plates for suspended cell cultures (3516, Corning, Somerville, MA, USA), sealed with parafilm to prevent evaporation, in 250 mL polycarbonate flasks for purified single colonies with vented caps (17211, Beijing Labgic Technology Co., Beijing, China). Symbiodiniaceae genomes were isolated using a Hi-DNA secure Plant Kit (TianGen Biotech Co. Ltd., Beijing, China) following the manufacturer’s user manual. See Table S3 for target gene primers and PCR cycle information.

Five microliters of each PCR product was run on 1% agarose gel to confirm successful amplification. PCR products were sequenced on a 3730xl DNA analyzer (Illumina PE150, San Diego, CA, USA) platform. The sequences were quality-trimmed using Sequencher 4.6 and analyzed by BLAST (https://blast.ncbi.nlm.nih.gov/Blast.cgi, accessed on 19 March 2025) of National Biotechnology Information Center. All sequences were deposited in the NCBI database under an individual accession number, given below (see Table S4).

### 2.3. Symbiodiniaceae–Bacteria Co-Culture

Bacteria were freshly plated before each experiment on MA2216E and cultured from single colonies in marine broth overnight (30 °C, 140 rpm). Following incubation, bacterial cells were centrifuged at 10,000× *g*, washed twice with sterile seawater, and re-suspended in a nitrogen-free L1 medium with specific absorbance levels at 600 nm (0.005, 0.01, 0.05, 0.1, and 0.5).

Symbiodiniaceae from early to mid-exponential phase cultures were inoculated into 200 mL of a nitrogen-free L1 medium to an initial cell density of 10,000 cells per milliliter. Cultures were supplemented with different concentrations of pre-cultured bacteria. Flasks were placed in an intelligent light incubator (PGX-680C, Safe, Ningbo, China) at 26 °C for 14 days, with a 12 h light/12 h dark cycle. 

### 2.4. N-Related Experimental Design and Sampling

The experiment was divided into four groups:

1. NP: A normal L1 medium with all elements sufficiently available.
2. NS: An L1 medium deficient in NaNO_3_.
3. NPD: An L1 medium deficient in NaNO_3_ co-cultured with *Pseudoalteromonas* sp. AH-5.
4. NB: An L1 medium deficient in NaNO_3_ co-cultured with *Bacillus* sp. AH-4.

The co-culture conditions were consistent with the previous setup, and physiological parameters of the cells were monitored over 14 days. Samples for omics analysis were collected on the final day. For sampling, the co-culture broth of bacteria and Symbiodiniaceae was filtered using a Buchner funnel device equipped with a 3 μm pore size polyethersulfone microporous filter membrane. Subsequently, the harvested Symbiodiniaceae biomass was subjected to two rounds of washing with sterile seawater, followed by a final filtration step to ensure purity and suitability for downstream analyses.

### 2.5. Physiological Index Detection of Symbiodiniaceae

Symbiodiniaceae growth was monitored by daily measurements of cell density and chlorophyll fluorescence, with photosynthetic pigments determined over 14 days. The cell number of Symbiodiniaceae was determined by direct counting under a microscope using a Sedgwick-Rafter counting chamber (3800, Saiji, Beijing, China), and abundance was calculated based on the sample volume.

Photochemical efficiency of AG11 cells was evaluated using pulse amplitude-modulated (PAM) fluorometry (Dual-PAM-100, WALZ, Effeltrich, Germany). To prevent non-photochemical dissipation of PS II excitation energy, samples remained in darkness for at least 30 min to ensure complete photochemical dissipation of the reaction centers. The maximum quantum yield of PS II photochemistry was determined as Fv/Fm.

Two milliliters of cell suspension was centrifuged at 6000× *g* for 7 min, supernatant discarded, and 2 mL of pre-cooled methanol (−4 °C) added to the pellet for photosynthetic pigment analysis. The mixture was incubated in darkness at 4 °C until the color of the pellet turned white. Absorbance of the supernatant was measured at 470, 665, and 720 nm.

Chlorophyll *a* (Chl *a*) and total carotenoid concentrations were calculated using the formulas below:

Chl *a* (μg/mL) = 12.9447 × (A_665_ − A_720_) (1)

Total carotenoids (μg/mL) = [1000 × (A_470_ − A_720_) − 2.86 × (Chl a (μg/mL))]/221 (2)

### 2.6. Transcriptome Sequencing and Data Analysis

RNA concentration and integrity were assessed using a Micro UV-Vis Spectrophotometer (NanoDrop 2000, Thermo Fisher, MA, USA) (and Agilent 2100/LabChip GX (Agilent, Santa Clara, CA, USA). Sequencing libraries were prepared using a NEBNext^®^ Ultra™ RNA Library Prep Kit for Illumina^®^ (NEB, Ipswich, MA, USA) following the manufacturer’s guidelines, with additional index codes for sample identification. PCR products were purified using the AMPure XP system (A63881, Beckman, Shanghai, China), followed by evaluation of library quality with the Agilent Bioanalyzer system (2100, California, USA). Indexed samples were then clustered on the cBot Cluster Generation System with the aid of a TruSeq PE Cluster Kit v3-cBot-HS (Illumina). Libraries were sequenced on an Illumina platform, generating paired-end reads. Raw data (fastq format) were processed using in-house Perl scripts. Q20, Q30, GC-content, and sequence duplication levels of the clean data were calculated, and all downstream analyses were relied on high-quality clean data. Differential expression analysis for the groups was conducted using the DESeq R package (1.10.1), with *p*-values adjusted by the Benjamini–Hochberg method to control the false discovery rate. Genes with adjusted *p*-values < 0.05 were classified as differentially expressed. Gene function was determined using the following databases: NR (for NCBI non-redundant protein sequences), Swiss-Prot (manually annotated and reviewed protein sequence database), Pfam (Protein family), KOG/COG/eggNOG (Clusters of Orthologous Groups of proteins), GO (Gene Ontology), and KEGG (Kyoto Encyclopedia of Genes and Genomes).

### 2.7. Metabolomics Profiling

Symbiodiniaceae cells were harvested on day 14, weighed, frozen in liquid nitrogen, and stored at −80 °C for further analysis. Samples were transferred to an EP tube in triplicates using 1000 μL of an extractant containing internal standards (methanol–acetonitrile, 1:1 *v*/*v*; internal standard concentration 20 mg L^−1^) and vortexed for 30 s. Samples were processed with a 45 Hz grinding instrument for 10 min, then by ultrasonic treatment in an ice-water bath for 10 min. Each sample was mixed into a QC (Quality Control) sample for machine detection.

Metabolite profiling analysis was performed using Waters Acquity I-Class PLUS UHPLC coupled with Waters Xevo G2-XS QTof high-resolution MS. Raw data collected with MassLynx V4.2 were processed using Progenesis QI software2.1 o determine peak extraction, peak alignment, and other data operations. After normalizing the original peak area information with the total peak area, follow-up analysis was conducted. Principal component analysis (PCA) was conducted to assess sample repeatability within groups and QC samples. Identified compounds were classified, and pathway information was retrieved from KEGG, HMDB, and lipidmaps databases. Differential metabolites were screened using fold change, *t*-test *p*-values, and VIP values from the OPLS-DA model. Screening criteria were fold change > 1, *p* < 0.05, and VIP > 1. Differential metabolite pathway enrichment significance was calculated using the hypergeometric distribution test.

### 2.8. Statistical Analysis

Statistical analyses were performed using *t*-tests and one-way analysis of variance (ANOVA) implemented in GraphPad Prism 10.0 software. Significance levels were defined as follows: “ns” (not significant) for *p* ≥ 0.05, * *p* < 0.05, ** *p* < 0.01, *** *p* < 0.005, and **** *p* < 0.001. All graphical representations, including histograms and line graphs, were generated using GraphPad Prism 10.0. Differential abundance heatmaps were constructed using R v3.1.1 with the pheatmap v1.0.2 package as the reference database.

## 3. Results

### 3.1. Symbiodiniaceae Growth with Coral-Associated Bacteria Enabled BMC Identification

**Figure 1:**
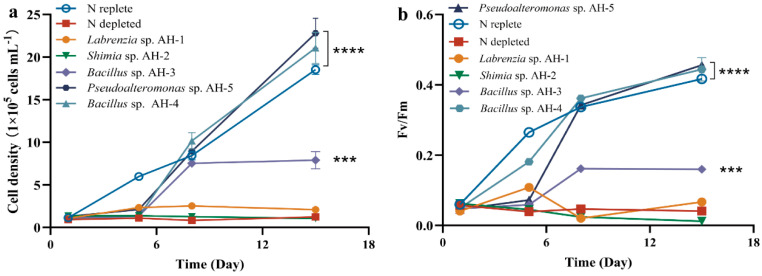
AG11 cell density (**a**) and Fv/Fm (**b**) changes over time under different culture conditions. Means ± SD for three independent trials are presented with the *p*-values (*t*-test) for the probabilities that the differences are significant. (*** *p* < 0.001; **** *p* < 0.0001).

From the initial collection of 42 isolated strains, five distinct species were selected for co-culture experiments: *Labrenzia* sp. AH-1, *Shimia* sp. AH-2, *Bacillus* sp. AH-3, *Bacillus* sp. AH-4, and *Pseudoalteromonas* sp. AH-5. AG11 was co-cultivated with five bacterial strains individually in a synthetic L1 medium, lacking inorganic nitrogen, ensuring that *Symbiodiniaceae* growth relied on bacteria-released organic molecules. The growth effect on AG11 significantly varied depending on the bacterial strain. No growth enhancement was found when AG11 was co-cultured with *Labrenzia* sp. AH-1 and *Shimia* sp. AH-2, which is consistent with nitrogen restriction. However, AG11 cell numbers increased over time when co-cultured with *Pseudoalteromonas* sp. AH-5 and *Bacillus* sp. AH-4, both of which significantly increased the specific growth rate of AG11 by 13.77–23.11% compared with the medium under N depleted condition. This suggests that these bacteria somehow modulate AG11 growth (Figure 1a). Meanwhile, *Bacillus* sp. AH-3 had a relatively weak effect on the promotion of cell density growth compared with *Bacillus* sp. AH-4 (Figure 1a). The Fv/Fm of AG11 showed a similar trend to cell density (Figure 1b).

Since *Pseudoalteromonas* AH-5 and *Bacillus* AH-4 consistently enhanced AG11 growth rate and these two strains are classified as beneficial coral bacteria [15,16], we further explored how bacterial and AG11 interactions improve survival under nitrogen limit conditions. Our assessment, validated by monitoring cell density and Fv/Fm in the co-culture, revealed that these metrics were greatly influenced by the bacterial inoculum size (Figure S1a–d). In N-deprived cultures, Fv/Fm and cell density remained low throughout the experiment. The Fv/Fm ratio with additional bacteria (OD_600_ = 0.5) was near 0.5 with *Bacillus* AH-4 and near 0.45 in cultures with *Pseudoalteromonas* AH-5. Higher bacteria concentrations led to increased activation rates (Figure S1b,d). Additionally, we evaluated the concentrations of chlorophyll and carotenoid in AG11 cultures grown under varying bacterial densities (Figure S1c,f). Both pigments are crucial for light energy absorption in photosynthesis. When bacterial optical density (OD_600_) reached 0.5, we observed a significant enhancement in the content of these photosynthetic pigments.

### 3.2. N-Restriction Triggers Global Response in AG11 Cells

**Figure 2:**
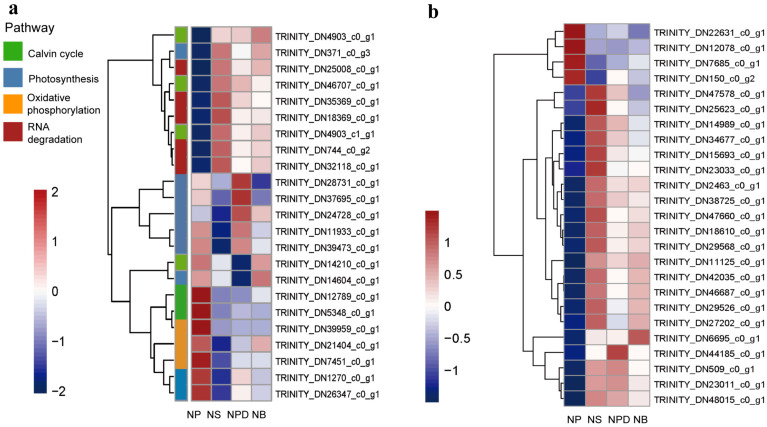
Heatmaps of the genome related to specific pathways. (**a**). Global response of AG11. (**b**). Genes related to N metabolism. The ordinate represents key differentially expressed genes; the abscissa represents different processing conditions. (Fold change ≥ 2, FDR < 0.01).

During N deprivation, Symbiodiniaceae cells trigger global responses that decelerate metabolism and redistribute nutrients through the degradation of protein and RNA. Concurrently, transcripts and proteins related to photosynthesis and the Calvin–Benson cycle decreased, leading to reduced photosynthetic capacity [17,18]. The principal component analysis (PCA) plot shows that the biological replicates of NS, NPD, and NB were clustered together, while the NPD replicates appeared to be more scattered. NB and NPD were significantly separated from NS and NP, indicating substantial transcriptomic differences between each group and suggesting distinct cellular response mechanisms under different culture conditions (Figure S2). Up-regulated genes in NS, NPD, and NB include CNOT1 (TRINITY_DN25008_c0_g1), PABPC (TRINITY_DN744_ c0_g2), and CNOT7/8 (TRINITY_DN18369_c0_g1, TRINITY_DN35369_c0_g1), which are associated with eukaryotic RNA degradation [19,20]. Enzymes involved in the Calvin–Benson cycle, such as Rubisco (TRINITY_DN14210_c0_g1), glyceraldehyde-3-phosphate dehydrogenase (TRINITY_DN12789_c0_g1), and fructose-bisphosphate aldolase (TRINITY_DN5348_c0_g1), were significantly down-regulated in the NS, NPD, and NB media. In addition, the light-trapping proteins LHCA1 (TRINITY_DN371_c0_g3) and the chlorophyll synthesis targeting enzyme glutamyl-tRNA reductase (TRINITY_DN21909_c0_g3) were inhibited under N-limiting conditions [21]. Co-culturing increased the abundance of transcripts associated with photosystem I (PsaE, TRINITY_DN1270 _c0_g1) and F-type ATPase (atpD, TRINITY_DN24728_c0_g1; atpA, TRINITY_DN39473_c0_g1); especially with Pseudoalteromonas sp., transcripts associated with photosystem II (psbD, TRINITY_ DN37695_c0_g1; psbC, TRINITY_DN11933_c0_g1) also increased (Figure 2a, Supplement Material S1).

### 3.3. Genetic Changes Associated with N Metabolism

Nitrate is a key nitrogen source in nature, but it needs to be reduced to ammonium levels for assimilation within the cell [22]. During N deprivation, AG11 cells induce a set of responses to facilitate N acquisition. There is a dramatic increase in the expression of specific ammonium transporters (AMTs, TRINITY_DN18610_c0_g1) that represent high transporters in NS, NPD, and NB groups (Figure 2b, Supplement Material S2) [23]. Microalgae take up inorganic nitrogen sources via nitrate and nitrite transporters [24]. Genes encoding the nitrite transporter (NAR, TRINITY_DN47578_c0_g1) were up-regulated in the NS, whereas no significant changes were observed in the NPD and NB. Genes encoding the nitrate transporter (NRT, TRINITY_DN12078_c0_g1) were up-regulated in NS, NPD, and NB groups. Nitrate reductase (NR, TRINITY_DN22631_c0_g1) activity has been reported to be inhibited when nitrate–nitrogen is deficient [25]; this is consistent with the decrease in NR that we detected in the experimental groups. Genes encoding nitrite reductases (NIR, TRINITY_DN23011_c0_g1), which plays a key role in the conversion of NO_2_^–^ to NH_4_^+^, are induced in N-limiting conditions. From this, we hypothesized that under nitrogen-deficient conditions, Symbiodiniaceae sense the external environment and release stored nitrate for the short-term maintenance of cellular activities. Genes encoding NADH-cytochrome b5 reductase (TRINITY_DN48015_c0_g1, TRINITY_DN509_c0_g1) and FAD-dependent oxidoreductase (TRINITY_DN42035_c0_ g1) were induced in NS, NPD, and NB groups, both of which are enzymes homologous to the sequence of the nitrate reductase structural domain and may also be involved in this process [26,27]. Meanwhile, Symbiodiniaceae also exhibit elevated organic N uptake as reflected by increased levels of transcripts encoding proteins involved in the transport and assimilation of purines (xanthine dehydrogenase, guanylate cyclase, TRINITY_DN46687_c0_g1) (Figure 2b, Supplement Material S2).

### 3.4. Metabolomic Analysis for Cellular Metabolic Reprogramming

**Figure 3:**
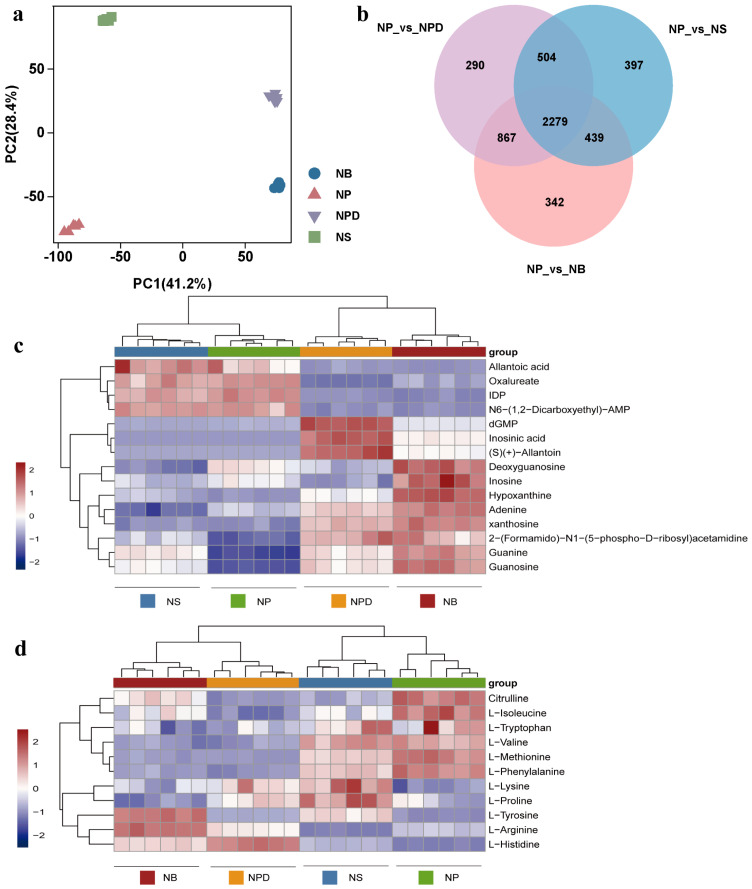
Principal component analysis (**a**), Venn diagram (**b**) and heatmaps of metabolites related to specific metabolic pathways. (**c**) Purine metabolic pathway. (**d**) Amino acid metabolic pathway. The ordinate represents key differentially expressed metabolites; the abscissa represents different processing conditions (fold change > 1, *p* < 0.05, and VIP > 1).

To further analyze metabolites involved in AG11 growth and development under different conditions, a widely targeted metabolome analysis was conducted using the LC-QTOF system. The PCA plot shows distinct separation among AG11 under the four conditions, with the six biological replicates for each condition clustering together (Figure 3a) and significant metabolite accumulation differences across conditions. Differential metabolites were identified using orthogonal partial least squares judgment, which extracted components from independent variable X and dependent variable Y to calculate correlations. To avoid model overfitting, 7-fold cross-validation and 200-time response permutation testing were performed, showing R^2^X values above 0.753. R^2^Y scores were 1, and Q^2^ values were greater than 0.998 for NP vs. NS/NPD/NB, indicating differential metabolite responses to treatments (Figure S3a–f). Venn plots highlight common and unique differences between metabolomes for both control and treatment groups, revealing 2279 common differential metabolites (Figure 3b). We analyzed the top 50 of these common differential metabolites (Figure S4, Supplementary Material S3), emphasizing inorganic nitrogen sources and bacteria’s role in enhancing metabolite accumulation, revealing specificity in metabolite accumulation among bacteria from distinct genera. These metabolites were categorized into organic acids and derivatives, lipids, carbohydrates, amino acids and derivatives, and organoheterocyclic compounds, focusing on amino acids and organic acids.

### 3.5. Co-Culture with Bacteria Induced Changes in Purine Metabolism Pathway

Nucleotides, nucleic acid hydrolysates, were up-regulated in NPD and NB groups: inosine monophosphate (IMP), 2′-deoxyguanosine5′-monophosphate (dGMP), guanosine, guanine, xanthosine, and hypoxanthine (Figure 3c). These metabolites participated in the purine metabolism pathway. Adenine and dGMP showed significant down-regulation under N stress. Compared with the NP group, inosine increased in the NB group but showed a down-regulated trend in the NPD group. Inosine 5′-diphosphate (IDP), deoxyguanosine, and adenylosuccinate displayed an overall down-regulation in NPD and NB groups (Figure 3c). Although guanosine and guanine accumulate more in NS than in NP, their accumulation was relatively smaller compared with NB and NPD.

### 3.6. Co-Culture with Bacteria Induced Changes in Amino Acid Biosynthesis

Under N stress, L-histidine and L-tyrosine were significantly up-regulated in NS, NPD, and NB compared with NP (Figure 3d). Concurrently, L-histidine exhibited significant enrichment in NB and NPD compared with NS, while L-tyrosine enrichment was exclusive to NB. In addition, nine amino acids and their derivatives were identified. L-phenylalanine, citrulline, L-methionine, and L-isoleucine showed a downward trend in NS, NPD, and NB, while L-valine was down-regulated in NB and NPD (Figure 3d). L-arginine was down-regulated in NS but up-regulated in NPD and NB compared with NP. L-proline was exclusively enriched in NS, with consistent concentrations in the remaining groups. L-lysine was enriched in NPD and NS, with no changes in the other groups. L-tryptophan concentrations remained consistent across all four groups. Overall, NP vs. NS, NP vs. NPD, and NP vs. NB showed a decreasing trend in amino acid accumulation, indicating preferential intracellular amino acid degradation by cells as an adaptive response to inorganic N deficiency, aiding their environmental adaptability.

### 3.7. KEGG Annotation Analysis of Key Metabolites and Genes

**Figure 4:**
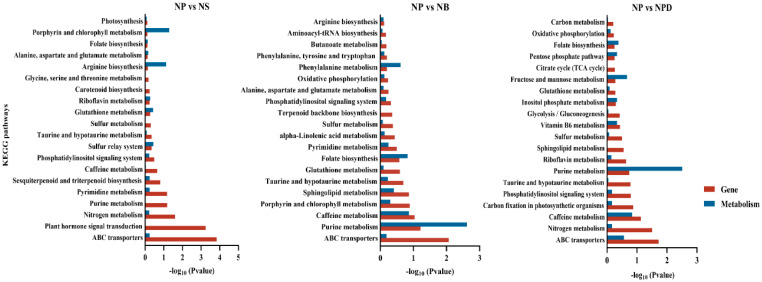
The KEGG enrichment plot of co-analysis of the transcriptome and metabolome of AG11 under different culture conditions. Differential pathway enrichment significance was calculated using the hypergeometric distribution test.

Based on KEGG pathway annotations, key differentially expressed proteins and metabolites from combined transcriptomic and metabolomic analyses were identified as being involved in pathways crucial to nutrient cycling and stress tolerance. These pathways include ABC transporters, amino acid, purine metabolism, nitrogen metabolism, glutathione and phosphatidylinositol metabolism, and caffeine metabolism (Figure 4). The annotation of these pathways indicates that algae possess intricate metabolic regulatory mechanisms involved in nutrient absorption, energy metabolism, antioxidant defense, signal transduction, and environmental adaptation.

## 4. Discussion

Culturing most dinoflagellates in vitro is challenging, and what can be cultured represents only a small fraction of the vast symbiotic Symbiodiniaceae–bacterial community. When the supply of exotic nutrients is low, it is expected that the growth of flagellates will benefit more from the regenerated nitrogen and phosphorus delivered by the bacteria. Nitrogen, a crucial nutrient, significantly affects the growth and development of microalgae. Nitrogen is vital for the free living of Symbiodiniaceae, and its absence adversely affects physiological indices [28]. To verify that beneficial BMC can provide the alternative nitrogen sources needed by Symbiodiniaceae, we first designed a nitrogen-limitation experiment for Symbiodiniaceae. This experiment demonstrated the essential requirement of nitrogen for the growth of Symbiodiniaceae. Subsequently, we added beneficial bacteria to the nitrogen-limited cultures of Symbiodiniaceae.

We assessed the health and nutrient status of the cultures by monitoring PSII efficiency and cell density for NP/NS [29]. It has been shown that *Chlamydomonas* cells slow down metabolism through the degradation of protein and RNA, and transcripts and proteins associated with the photosynthesis and the Calvin–Benson cycle decline during the N deprivation, leading to diminished photosynthetic capacity [17]. This is consistent with our detection of increased levels of CNOT7/8, CNOT1, and PABPC transport proteins associated with RNA degradation in Symbiodiniaceae during N deprivation. Additionally, a decrease in intracellular enzymes related to the photosynthesis (LHCA1, glutamyl-tRNA reductase) and Calvin cycle (Ribisco, GAP3, and FBA) suggests that Symbiodiniaceae might extract nitrogen from N-rich chloroplast proteins under prolonged nitrogen starvation [30]. However, while nitrogen uptake and translocation can temporarily sustain Symbiodiniaceae cells, they do not allow for sustained division and growth. These transporters facilitate the uptake and assimilation of these nitrogen forms, enabling the symbionts to maintain essential physiological functions despite the scarcity of inorganic nitrogen source in the medium [31]. This regulatory mechanism underscores the symbionts’ ability to dynamically adjust to varying nitrogen availability, ensuring continued photosynthetic efficiency and overall metabolic stability [32].

Symbiodiniaceae–bacteria interactions are complex, involving various molecular signals, metabolites, transporters, and molecules whose functions remain under-explored [30,33]. In our study, we tested 5 coral-associated bacterial strains in a N-limited medium; 3 of them did not exhibit any positive effects on Symbiodiniaceae cell cultures. Although the remaining two strains, *Pseudoalteromonas* AH05 and *Bacillus* AH01, demonstrated enhancement of the algal growth, the former seemed to be more effective than the latter. Recent studies have shown high host specificity among bacterial communities associated with Symbiodiniaceae, with bacterial species and strains performing similar metabolic functions colonizing similar algal taxa [34]. This aligns with our findings of different cultivation results depending on the strains of Symbiodiniaceae used.

In co-culture, AG11 increased transcripts associated with ammonium transport and altered purine metabolism, indicating coordinated expression of cellular pathways utilizing alternative nitrogen sources. The AMT family, encoding ammonium transporters and localized in plasma and chloroplast membranes, displays coordinated up-regulation in response to a nitrogen-free medium [23]. Symbiodiniaceae also facilitate nitrogen acquisition by up-regulating nitrate transporter and nitrite reductase genes, promoting inorganic to organic nitrogen conversion [17]. Moreover, transcriptional expressions for NPD and NB were up-regulated in guanine, adenine, and hypoxanthine metabolism compared with NP and NS, with XDH gene up-regulation and the production of uric acid providing an organic nitrogen source [35,36]. Purine metabolism offer a continuous nitrogen source and endogenous pathway for nitrogen reutilization in plant growth [37], with efficient conversion of xanthine into active catabolic metabolites crucial for normal growth. For instance, the ability of *Chlamydomonas* to utilize xanthine and hypoxanthine as a N source represents a significant metabolic adaptation, particularly when ammonia or nitrates are absent from the environment; *Chlamydomonas* relies heavily on XDH to scavenge for alternative N sources, thereby sustaining its growth and survival [17,38]. Under identical N-deficient conditions, the overexpression of *OsUPS1*, the gene encoding the ureide permease in rice, significantly enhances plant growth via the augmented translocation of xanthine-derived catabolic metabolite allantoin into plant tissue [33,39]. These results suggest that, in co-culture, AG11 enhances ammonium transport and purine uptake, up-regulating transcripts associated with photosynthesis, indicating the essential role of reduced nitrogen in the interaction.

**Figure 5:**
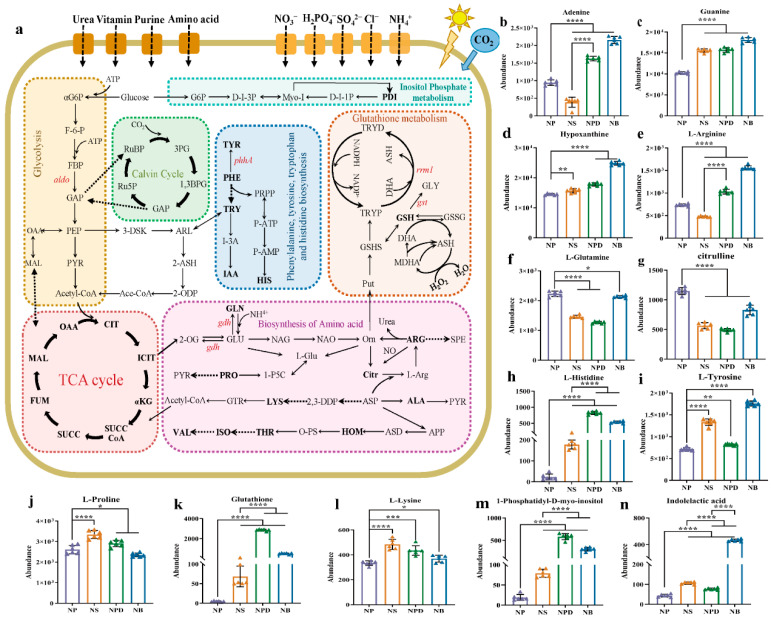
(**a**) Screening for maps of metabolic pathways involved in key differentially expressed metabolites. The pathway map includes mainly glycolysis, TCA cycle, Calvin cycle, biosynthesis of amino acids, glutathione metabolism, and inositol phosphate metabolism. Full name corresponds to the abbreviation in the Supplementary Materials. (**b**–**n**) Cylindrical chart of the content changes of 13 differentially expressed metabolites. Data are presented as the mean and SD. Statistical analysis was performed using the Student’s *t*-test. (n = 6, * *p* < 0.05; ** *p* < 0.01; *** *p* < 0.001; **** *p* < 0.0001).

**Figure 6:**
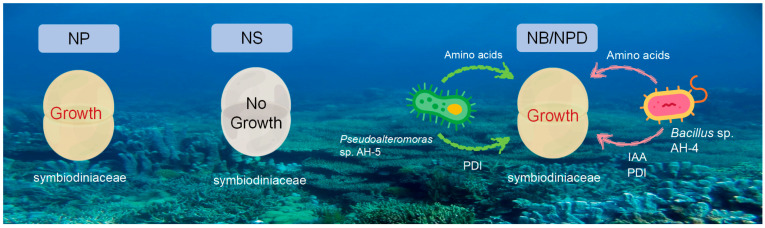
Comparison of Symbiodiniaceae growth under different conditions: 1. NP group: in N^+^ replete medium; 2. NS group: in N^+^ deplete medium; 3. NB/NPD groups: in N^+^ deplete medium, with addition of beneficial bacteria that provide nitrogen source and other metabolites to sustain Symbiodiniaceae cultures.

Amino acids have been identified among the main mediator molecules that regulate the relationship between microalgae and bacteria. Amino acid biosynthesis is vital for the growth and development of organisms. In nature waters, a large number of microalgae have been found to use amino acids and other organic N sources in addition to inorganic N [40,41]. Under co-culture conditions, we detected a decrease in glutamine and enrichment of L-arginine. Glutamate and glutamine feed into the urea cycle, while L-arginine and citrulline are cycle components (Figure 5). Co-culture with bacteria increased AG11 transcripts associated with the arginine pathway, converting and dissolving L-arginine into polyamines (spermine and spermidine), which play crucial roles in maintaining plant growth and development [42,43]. The accumulation of histidine in cells may be associated with the biosynthesis of purine mononucleotides, and the relationship between them has been recognized from genetic studies of bacteria and yeast [44]. In co-culture, we detected a high concentration of histidine, indicating that histidine metabolism may also be a coordinated “nutritional supplement” pathway. Studies have shown that in the presence of histidine as the sole source of N, cell growth was comparable with that observed under the same concentration of ammonium nitrogen [45]. Inside the algal cells, histidine can be degraded into glutamate, and to a certain extent, it can directly replenish energy for metabolic pathways [41]. In addition to the involvement of arginine and histidine in the cellular metabolic cycle of AG11, in the case of energy deficiency, L-lysine catabolism fluxes directly into the TCA cycle [46]. Proline is a proteogenic amino acid that participates in the biosynthesis of primary metabolic products, regulates osmosis, and protects proteins under stress conditions [47,48]. The accumulation of proline and lysine was detected in co-culture with *Pseudoalteromonas*, indicating that these two amino acids can also provide nitrogen nutrition for AG11, ensuring normal growth. Tyrosine serves as a central hub for numerous specialized metabolic pathways and also a precursor for various specialized metabolites with distinct physiological functions, such as non-protein amino acids, attractants, and defense compounds [49]. During the co-cultivation of Symbiodiniaceae with Bacillus, the accumulation of tyrosine was detected, indicating that tyrosine is also a nutrient for the growth of AG11 cells, while we are unable to distinguish whether the compounds were directly translocated from the bacteria or were downstream metabolites; the enrichment of these compounds in Symbiodiniaceae suggests that the translocated compounds or products were utilized by Symbiodiniaceae and entered their urea cycle (Figure 6). The nitrogen cycle involves ammonium production through the urea cycle, promoting algal replication. Amino acid metabolism closely relates to energy and carbohydrate metabolism, carbon–nitrogen budgeting, protein synthesis, and secondary metabolism. The importance of amino acid in benthic diatoms and bacteria interactions has recently also been recognized in natural phyllosphere biofilm. In benthic diatom *Phaeodactylum tricornutum*/*E. coil* co-cultures, extracellular dissolved free amino acid concentrations varied based on diatom and bacteria strain combinations [50]. These results suggest that, under inorganic nitrogen deficiency, cells may be “nutrient-compensated” by up-regulating arginine and lysine-related pathways to maintain normal cell division and value addition.

Bacteria can produce hormones like indole-3-acetic acid (IAA), cytokinins, myo-inositol, and gibberellins, which play crucial roles in modulating algal growth, nutrient uptake, and stress responses [51]. A recent study reveals that myo-inositol serves as the main substrate for synthesizing phosphatidylinositol and phosphatidylinositides, which function as mediators of intercellular and intracellular communication during cellular growth and play a pivotal role in responding to environmental changes [52]. In NPD and NB, the *myo*-inositol metabolite phosphatidyl-1D-*myo*-inositol (a kind of phosphatidylinositol, PDI) accumulated in large quantities in cells, whereas small amounts of PDI were maintained in NS and NP, suggesting that bacteria increased the utilization of intracellular *myo*-Inositol in co-culture, potentially contributing to the promotion of microalgal growth by triggering multiple sensors and simultaneously acting as a communication medium linking diverse signaling pathways [53]. IAA, a form of auxin, influences cell elongation and division, enhancing nutrient assimilation and growth in Symbiodiniaceae. For example, *Sulfitobacter* promotes the growth of diatoms by secreting IAA synthesized from diatom-derived tryptophan, demonstrating that the mutual exchange of metabolites affects the success of phytoplankton partners [54]. The addition of IAA to the cultures of the diatom *Skeletonema costatum* caused an increase in the number of cells in a chain [55]. Co-culture with *Bacillus* sp. AH-4 triggered the accumulation of IAA in AG11 (Figure 6), an endogenous plant hormone that is also produced and excreted by rhizobia to skew symbiotic plant development [56]. The large enrichment of IAA only in the NB group suggests that the growth hormone is not secreted and available to cultures by all bacteria, and that the binding of specific bacteria and algae is required to detect the enrichment of IAA.

Cells produce and accumulate large amounts of ROS internally during nitrogen stress, leading to oxidative stress on many intracellular structures and components causing cellular damage [57,58]. Glutathione (GSH) and glutathione-related enzymes play a crucial role in determining the tolerance of a plant under various stresses and can participate in the scavenging of ROS formed during redox cycling. We found that GSH and its downstream metabolites in the N-limited group significantly accumulated in AG11 cells, especially in the NB, suggesting that the cells are subjected to oxidative stress during the transfer from N-filled to N-limited. In addition, the GSH metabolic cycle regenerates ascorbic acid (ASH), another potential water-soluble antioxidant [59]. Agarwal reported that the GSH and ASH contents were significantly increased by the two doses of UV-8 stress in *Cassia auriculata* seedlings to protect tissue from ROS [60]. In summary, under inorganic N limitation conditions, cells up-regulate the glutathione metabolic pathway to eliminate reactive oxygen species within the cell, thus ensuring the normal functioning of internal organs.

Our research provides a theoretical foundation to support coral reef restoration and the sustainability of reef ecosystems. Key strategies include introducing probiotics to enhance coral health, utilizing diazotrophs to address nitrogen limitation, and developing microbiome-based restoration plans. Ecological modeling incorporates nitrogen fixation and microbial interactions to predict reef stability under environmental stress. Together, these efforts bridge science and conservation, promoting resilient and sustainable coral reef ecosystems.

## 5. Conclusions

This study provides a molecular perspective on the bacteria–Symbiodiniaceae association, highlighting specific nitrogen metabolic processes that may coordinate the trophallaxis of partner organisms to provide alternative nitrogen source to sustain Symbiodiniaceae cell growth. Our observation revealed a significantly elevated growth rate of Symbiodiniaceae, whether freely living in the water or associated with beneficial bacteria. This suggests that the probiotic bacteria furnish critical metabolic resources to support the successful proliferation of dinoflagellates outside the hosts, potentially serving as “resource surrogates”. However, symbiotic bacteria do not uniformly exhibit identical behaviors, and strains of the same or different genera may yield diverse outcomes.

## Data Availability

The original contributions presented in this study are included in the article/Supplementary Material. Further inquiries can be directed to the corresponding author.

## Supplementary Materials

The following supporting information can be downloaded at: https://www.mdpi.com/article/10.3390/microorganisms13040748/s1, Figure S1. AG11 cell density, chlorophyll fluorescence, and photosynthetic pigment content based on different bacterial concentration. (a–c) Co-culture with *Pseudoalteromonas* AH-5; (d–f) Co-culture with *Bacillus* AH-4. Means ± SD for three independent trials are shown with the *p*-values (*t*-test) for the probabilities that the differences are significant. (**** *p* < 0.0001). Figure S2 Principal component analysis (a), venn diagram (b) of differential genome of AG11 under different culture conditions. Figure S3 Orthogonal partial least squares discriminant analysis of differential metabolites of AG11 under different culture conditions. Positive mode (a–c). negative mode (d–f). Figure S4. Metabolite clustering heatmap of the top 50 common differential metabolites. Table S1. The components of the L1 medium. Table S2. The components of the Marine Agar 2216E medium. Table S3. Target gene primers and PCR cycle information. Table S4. Comparison of sequence matches from BLAST searches using 16S rDNA genes of bacteria. Supplement Materials S1: Table S5. Gene annotation and KEGG enrichment of global metabolic pathways of Symbiodiniaceae AG 11. Supplement Materials S3: Table S6. Gene annotation and KEGG enrichment of nitrogen metabolic pathways of Symbiodiniaceae AG 11. Supplement Materials S3: Table S7. Top 50 commonly existing metabolites among the four groups (NP, NS, NPD and NB) in Symbiodiniaceae AG11.
