# Supplementary material for: Coral-Associated Bacteria Provide Alternative Nitrogen Source for Symbiodiniaceae Growth in Oligotrophic Environment
Source: Microorganisms. 2025 Mar 26;13(4):748. doi: 10.3390/microorganisms13040748 (PMC12029909; doi:10.3390/microorganisms13040748)
Supplement: Supplementary file 1 [file microorganisms-13-00748-s001.zip › microorganisms-3526518-supplementary.pdf]

Supplementary Materials

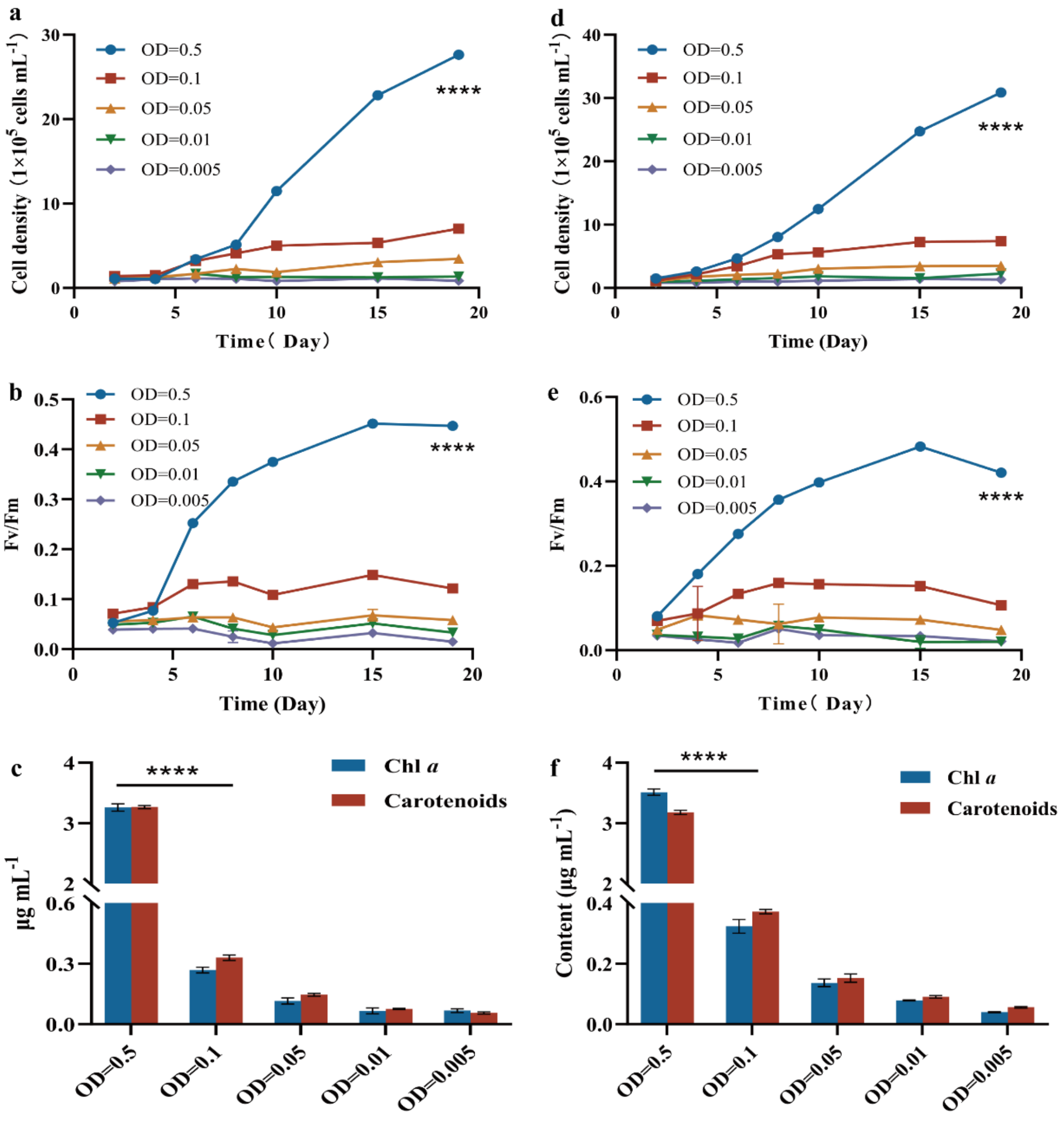

Figure S1. AG11 cell density, chlorophyll fluorescence, and photosynthetic pigment content based on different bacterial concentration. (a-c) Co-culture with *Pseudoalteromonas* AH-5; (d-f) Co-culture with *Bacillus* AH-4. Means ± SD for three independent trials are shown with the p-values (t-test) for the probabilities that the differences are significant. (**** $p < 0.0001$)

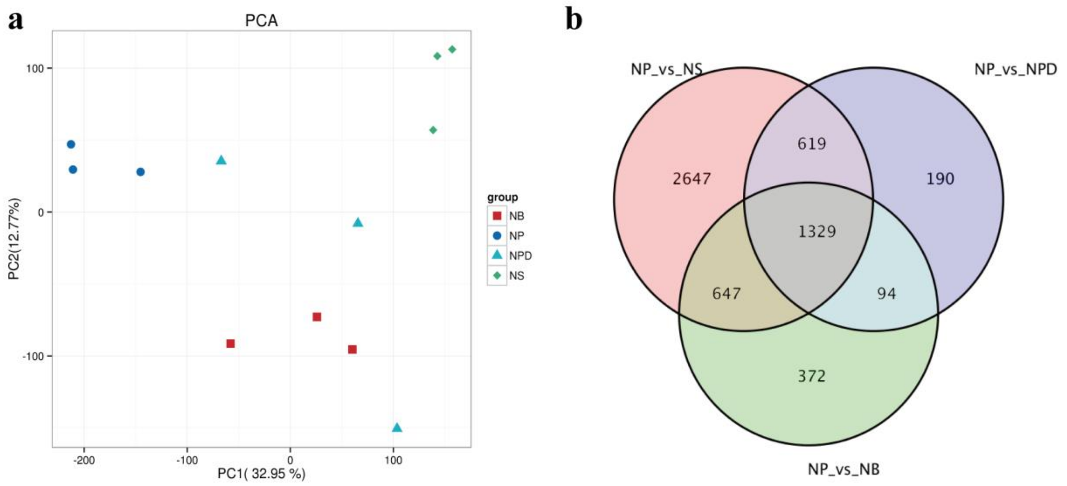

Figure S2 Principal component analysis (a), venn diagram (b) of differential genome of AG11 under different culture conditions.

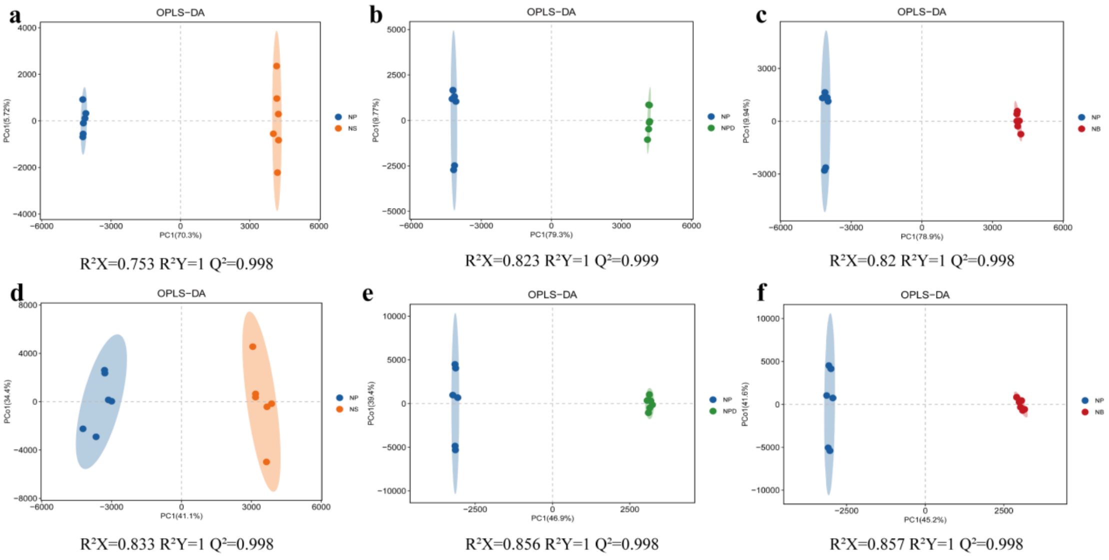

Figure S3 Orthogonal partial least squares discriminant analysis of differential metabolites of AG11 under different culture conditions. Positive mode (a-c). negative mode (d-f).

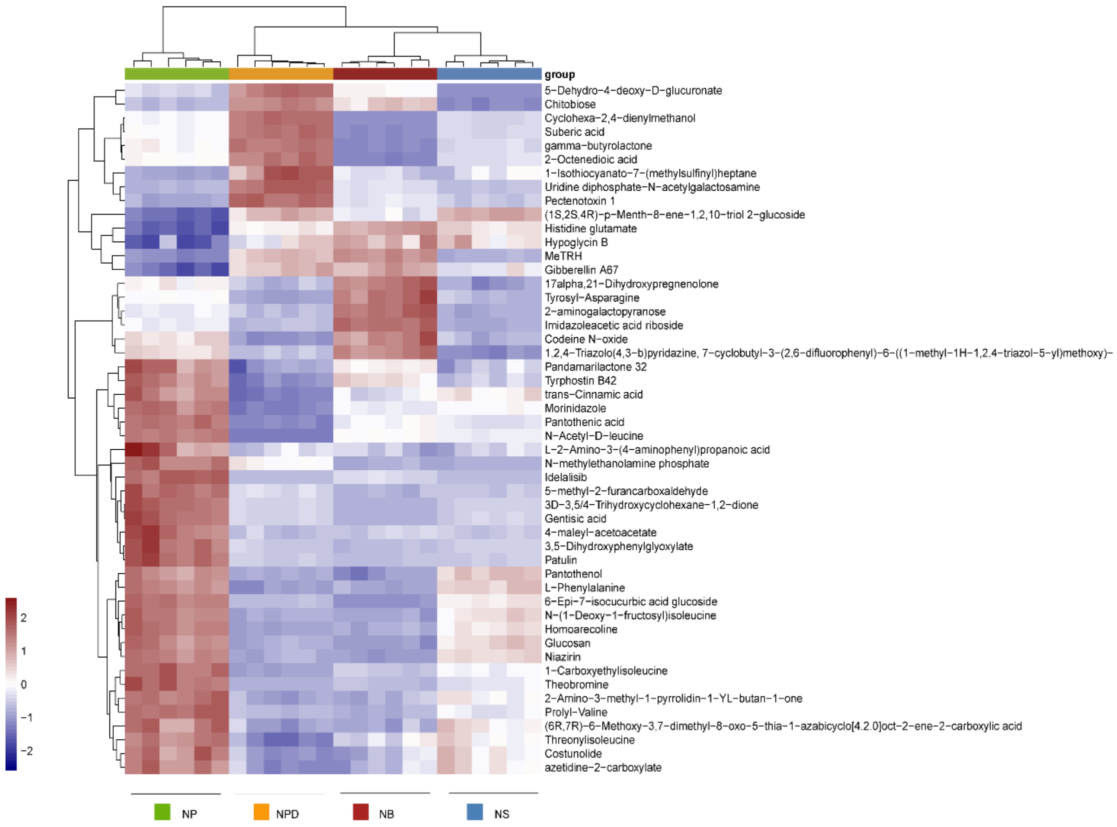

Figure S4. Metabolite clustering heatmap of the top 50 common differential metabolites

Abbreviations in Fig. 5a against full name:

Inositol Phosphate metabolism: D-I-3P: 1D-myo-Inositol-3p; Myo-I: myo-Inositol; D-I-1P: 1D-myo-Inosiyol-1P; PDI: phosphatidyl-1D-myo-inositol;

Biosynthesis of Amino acid: 2-OG: 2-Oxoglutarate; GLU: Glutamate; GLN: Glutamine; NAG: N-Acetyl-glutamate; NAO: N-Acetylornithine; Orn: Ornithine; Arg: Arginine; Citr: Citruline; L-Arg: L-Arginosuccinate; L-Glu: L-Glutamate 5-semialdehyde; SPE: Spermine; 1-P5C: 1-Pyrroline -5-carboxylate; PRO: Proline; PYR: Pyruvate; ASP: Aspartate; ALA: Alanine; 2,3-DDP: 2,3-Dihydrodipicolinate; LYS: Lysine; GTR: Glutarate; APP: Aspartyl phosphate; ASD: Aspartyl semialdehyde; HOM: Homoserine; O-PS: O-phosphohomoserine; THR: Threonine; ISO: Isoleucine; VAL: Valine

Glutathione metabolism: Put: Putrescine; GSHS: Glutathionyspermidine; GSH: Glutathione; TRYP: Trypanothione; TRYD: Trypanothionedisulfide; DHA: Dehydroascorbate; ASH: Ascorbate;

Phenylalanine, tyrosine, tryptophan and histidine biosynthesis: TRY: tryptophan; TYR: tyrosine; PHE: phenylalanine; I-3A: Indole-3-acetamide; IAA: Indoleacetate; PRPP: 5-Phosphoribosyl diphosphate; P-ATP: Phosphoribosyl-ATP; P-AMP: Phosphoribosyl-AMP; HIS: Histidine

Glycolysis: αG6P: α-D-Glucose 6-phosphate; F-6-P: D-Fructose 6-phosphate; FBP: D-Fructose 1,6-bisphosphate; GAP: D-Glyceraldehyde 3-phosphate; PEP: Phosphoenolpyruvate; OAA: Oxaloacetate; MAL: Malate;

TCA cycle: CIT: citrate; ICIT: Isocitrate; SUCC CoA: Succinyl-CoA; SUCC: Succinate; FUM: Fumarate;

Calvin Cycle: RuBP: D-Ribulose 1,5-bisphosphate; Ru5P: D-Ribulose 5-phosphate; 3PG: 3-phosphor-D-glycerate; 1,3BPG: 1,3-bisphospho-D-glycerate

3-DSK: 3-Dehydroshikimate; ARL: Anthranilate; 2-ASH: 2-Aminomuconate Semialdehyde; 2-ODP: 2-Oxoadipate;

Table S1

The components of the L1 medium.

	Components	ultimate concentration
	$NaNO_3$	75 μg L^{-1}
	$NaH_2PO_4 \cdot H_2O$	5 μg L^{-1}
trace element solution	$Na_2EDTA \cdot 2H_2O$	4.36 μg L^{-1}
	$FeCl_3 \cdot 6H_2O$	3.15 μg L^{-1}
	$MnCl_2 \cdot 4H_2O$	178.10 μg L^{-1}
	$ZnSO_4 \cdot 7H_2O$	23.00 μg L^{-1}
	$CoCl_2 \cdot 6H_2O$	11.90 μg L^{-1}
	$CuSO_4 \cdot 5H_2O$	2.50 μg L^{-1}
	$Na_2MoO_4 \cdot 2H_2O$	19.90 μg L^{-1}
	H_2SeO_3	1.29 μg L^{-1}
	$NiSO_4 \cdot 6H_2O$	2.63 μg L^{-1}
	Na_3VO_4	1.84 μg L^{-1}
	K_2CrO_4	1.94 μg L^{-1}
vitamin solution	Vit.B_1	2.96×10^{-7} M
	Vit.H	2.05×10^{-9} M
	Vit.B_{12}	3.69×10^{-10} M

Table S2

The components of the Marine Agar 2216E medium.

Components	ultimate concentration
NaCl	19.45 g L^{-1}
$MgCl_2$	8.8 g L^{-1}
Na_2SO_3	3.24 g L^{-1}
$CaCl_2$	1.8 g L^{-1}
KCl	0.55 g L^{-1}
$NaHCO_3$	0.16 g L^{-1}
$FeCl_2$	0.1 g L^{-1}
KBr	0.08 g L^{-1}
$SrCl_2$	0.03 g L^{-1}
H_3BO_3	0.02 g L^{-1}
tryptone	5 g L^{-1}
yeast extract	1 g L^{-1}

Table S3

Target gene primers and PCR cycle information.

Target gene	Forward primer sequence	Reverse primer sequence	Thermocycling
ITS2 rDNA	5′- ATC GAT GAA GAA CGC AGC - 3′	5′- TCC TCC GCT TAT TGA TAT GCC CCG - 3′	95 ℃ for 3 min; 30 cycles of 95 ℃ for 30 s, 53 ℃ for 30 s, and 72 ℃ for 2 min; and final extension cycle of 5 min at 72 ℃
16S rRNA	27F (5′-AGA GTT TGA TCA TGG CTC AG -3′)	1492R (5′-GTT TAC CTT GTT ACG ACT T -3′)	94 ℃ for 3 min; 30 cycles of 94 ℃ for 40 s, 55 ℃ for 1 min, and 72 ℃ for 2 min; and final extension cycle of 10 min at 72 ℃

Table S4

Comparison of sequence matches from BLAST searches using 16S rDNA genes of bacteria.

No.	Genus	Name	Accession Number	Identity (%)
AH-1	*Roseobacteraceae*	*Shimia* sp. strain R9_1	OR835779.1	99.92
AH-2	*Labrenzia*	*Labrenzia* sp. strain CAU 1498	OR835782.1	99.85
AH-3	*Bacillus*	*Bacillus weihaiensis*	OR835768.1	100
AH-4	*Bacillus*	*Bacillus coahuilensis*	OR835769.1	99.28
AH-5	*Pseudoalteromonas*	*Pseudoalteromonas* sp. CF6-2	OR835791.1	100
AG11	*Durusdinium* sp.	*Durusdinium* sp.	OR835803.1	99
